# Chirality and odd mechanics in active columnar phases

**DOI:** 10.1093/pnasnexus/pgae398

**Published:** 2024-09-10

**Authors:** S J Kole, Gareth P Alexander, Ananyo Maitra, Sriram Ramaswamy

**Affiliations:** Centre for Condensed Matter Theory, Department of Physics, Indian Institute of Science, Bangalore, Karnataka 560 012, India; INI, University of Cambridge, Cambridge CB3 0EH, United Kingdom; DAMTP, Centre for Mathematical Sciences, University of Cambridge, Cambridge CB3 0EH, United Kingdom; Department of Physics, University of Warwick, Coventry CV4 7AL, United Kingdom; Laboratoire de Physique Théorique et Modélisation, CNRS UMR 8089, CY Cergy Paris Université, Cergy-Pontoise Cedex F-95032, France; Laboratoire Jean Perrin, Sorbonne Université and CNRS, Paris F-75005, France; Centre for Condensed Matter Theory, Department of Physics, Indian Institute of Science, Bangalore, Karnataka 560 012, India; International Centre for Theoretical Sciences, Tata Institute of Fundamental Research, Bangalore, Karnataka 560 089, India

**Keywords:** chiral liquid crystal, active matter, columnar liquid crystals

## Abstract

Chiral active materials display odd dynamical effects in both their elastic and viscous responses. We show that the most symmetric mesophase with 2D odd elasticity in three dimensions is chiral, polar, and columnar, with 2D translational order in the plane perpendicular to the columns and no elastic restoring force for their relative sliding. We derive its hydrodynamic equations from those of a chiral active variant of model H. The most striking prediction of the odd dynamics is two distinct types of column oscillation whose frequencies do not vanish at zero wavenumber. In addition, activity leads to a buckling instability coming from the generic force-dipole active stress analogous to the mechanical Helfrich–Hurault instability in passive materials, while the chiral torque-dipole active stress fundamentally modifies the instability by the selection of helical column undulations.

**Significance Statement**

We establish minimal ingredients for the emergence of odd elasticity and odd viscosity—certain asymmetries in the relation of stress to strain and strain-rate—in a 3D fluid. The resulting structure is that of a columnar liquid crystal with polar and chiral active stresses. Predicted outcomes include oscillatory dynamics without mechanical inertia, with viscous hydrodynamics leading to a frequency that remains nonzero in the limit of infinite wavelength, in marked contrast to 2D odd elasticity. These oscillations should be seen in vitro in biofilament-motor complexes and are likely to arise in living columnar structures such as axons and muscle.

## Introduction

Living matter continually converts chemical energy into work. A description of mechanics and statistics of such microscopically driven materials must either explicitly account for this chemistry (1, 2) or introduce a parameter that breaks time-reversal symmetry at the microscopic level (3–8). This latter description, termed active matter, has been used to describe the novel dynamics, mechanics, and statistics of a plethora of broken-symmetry phases (9–16). In chiral (17, 18) 2D solids, an active mechanical effect that is the subject of much current attention is “odd elasticity” (13, 15, 19), a generalized relation between stress and strain, breaking a fundamental symmetry or reciprocity that holds in thermal equilibrium. This phenomenon was recently highlighted in a setting related to developmental biology (20) though anticipated in part in early studies on rotating Rayleigh–Bénard convection (21, 22). A key feature is the existence of elastic oscillations in a regime in which mechanical inertia is manifestly negligible. This odd dynamics results from a ratio of elastic and viscous coefficients and, in fact, can arise in two distinct ways: as a ratio of odd elasticity to even viscosity or of even elasticity to odd viscosity. In this article, we show how odd coefficients emerge naturally through spontaneous translation symmetry-breaking in an active system, in concert with two discrete asymmetries: *chirality*, i.e. the impossibility of superimposing individual units or structures on their mirror images (18), and *polarity*, a vector orientation or lack of inversion symmetry. We will do this in the spirit of the classical treatments (23–26) that yield the elastic properties of a solid as a density wave in a fluid. This requires augmenting model H (27) to include active processes (28, 29), chirality (12), and polar symmetry breaking. The 3D odd viscosity—a contribution to the tensor connecting stress and strain *rate* antisymmetric under an exchange of the first and second pair of indices (30, 31)—that then naturally arises also plays an important role in our treatment.

The long-range effects of active stresses emerge through fluid flow, whereas odd elasticity is a property of solids in two or more dimensions. Odd dynamics also arises in 3D chiral active mesophases with spatially modulated order (12, 21), where the elasticity of the spatial modulation couples to Stokesian hydrodynamics along any fluid directions. We present a study of the most symmetric system—a polar and chiral active columnar phase (32–35) with 2D translational order and one fluid direction—possessing odd elasticity in three dimensions. Our article is the first exploration of columnar materials in active systems, despite the abundance of filamentous assemblies in soft matter and biology (36–39). Not only is chirality the rule in biological matter, the monomeric units of most biopolymers are noncentrosymmetric. In particular, the microtubule bundles comprising the axon in nerve cell are a realization of active, columnar, chiral, macroscopically polar matter (40, 41). Columnar packings of DNA (37, 42, 43), if rendered active by transcription, are another candidate.

Our main result is the prediction of elastic oscillations in chiral and polar active columnar phases in an inertialess regime. Despite columnar materials breaking 3D rotation symmetry and 2D translation symmetry, the slow dynamics of the structure is governed by the elastic displacement of the columns. This comes from a combination of elastic forces and Stokesian friction coming from the conserved momentum density. In all systems with translational order, the low Reynolds number dynamics of the displacement field reads ${\dot{u}}_{q}=M_{q}\cdot F_{q}$.^a^ For modes with wavenumber *q*, the mobility $M_{q}$ scales as $1/q^{2}$, due to the long-range nature of the Stokes flow while the elastic force density $F_{q}$ scales as $∼q^{2}$, yielding a characteristic relaxation rate independent of the magnitude of the wavevector q=|q| for most directions. However, in equilibrium lamellar or columnar systems, there is no elastic response for material deformations with wavevector in liquid directions. In analogy with the lamellar case (10, 12, 16, 44), activity creates a column tension, and hence a nonvanishing q=0 response, even for q purely along the columns. In contrast to the lamellar case, active columnar materials display an emergent nonreciprocal dynamics of $u_{⊥}\equiv (u_{x},u_{y})$, a consequence of the odd elastic and viscous response of chiral polar systems. Each embodies nonreciprocity individually, through $F_{q}$ and $M_{q}$, respectively; the effective nonreciprocal dynamics, in the form of oscillatory modes due to the two components of the displacement field behaving like a position–momentum pair, emerges by combining the nonreciprocal part of one with the reciprocal part of the other. The two ways of doing this—${M_{q}}_{\mathrm{even}}\cdot {F_{q}}_{\mathrm{odd}}$ and ${M_{q}}_{\mathrm{odd}}\cdot {F_{q}}_{\mathrm{even}}$—have physically distinct dynamical signatures. Not only the “even–even” but also the “odd–odd” combinations contribute to the reciprocal response. Importantly, because of the long-range nature of the Stokesian dynamics, the oscillation frequency of this mode does not vanish in the limit of zero wave*number*. The frequency and the very existence of this odd collective oscillation, however, does depend on the angle between q and the column axis. Indeed, the 3D character of the columnar material is essential: incompressibility forbids this mode for in-plane perturbations.

Our additional predictions include a fundamental buckling instability driven by an apolar (force dipole) active stress in common with the lamellar case (12); in sharp contrast, the effect of chiral (torque dipole) active stresses is to remodel the structure. We now present in detail the theory from which these results follow.

## A polarizable chiral active suspension

We consider a bulk, 3D, two-component system with 3D scalar number density *ψ* of active polar and chiral units and momentum density g=ρv, as functions of position r=(x,y,z) and time *t*, with overall incompressibility: a constant total mass density *ρ* and ∇⋅v=0 for the joint velocity field v. The degree of vectorial alignment of the particles is accounted for by the polar order parameter field P. *ψ* obeys the conserving dynamics:


(1) $$\partial_{t}\psi =-\nabla \cdot (\psi v)+M\nabla^{2}\frac{\delta F}{\delta \psi },$$

where *F* is the free energy that would control the dynamics in the absence of activity; we discuss its form after constructing the dynamical equations for the velocity and the polarization fields.

We consider systems in which the Reynolds number is small at the scales of interest, as is the case for typical microbial and soft matter systems. In this limit, the velocity field is governed by the Stokes equation, with viscous forces balancing active and passive forces in the suspension:


(2) $$\nabla \cdot (\eta \nabla v)=-\nabla \psi \frac{\delta F}{\delta \psi }+\nabla \Pi -\nabla \cdot \sigma^{a},$$

where η is the viscosity tensor whose general form we will discuss in later sections, Π is the pressure that enforces the incompressibility constraint ∇⋅v=0, and $\sigma^{a}$ is the active stress. We ignore additional passive force densities involving P and δF/δP (45) whose effects in the polar columnar phase are subdominant to the active terms that we consider.

The expression of active stress in terms of *ψ* and P has three parts, all leading to terms at the same order in gradients in the polar columnar phase:


(3) $$\sigma_{ij}^{a}=-\zeta_{H}\partial_{i}\psi \partial_{j}\psi +\zeta_{\;pa}P_{i}P_{j}-{\bar{\zeta }}_{\;pc}(P_{l}\partial_{k}\psi \epsilon_{ikl}\partial_{j}\psi {)}^{S},$$

where the superscript *S* denotes symmetrization on the free indices, and $\epsilon_{ijk}$ is the Levi–Civita tensor. The first term is familiar from active model H (12, 28, 29, 46). The second is the standard active stress for liquid crystals (5, 47–49). The third, with coefficient ${\bar{\zeta }}_{\;pc}$, requires both chirality and polarity and is fundamentally biaxial. A force density with the symmetry of ${\bar{\zeta }}_{\;pc}$ has not hitherto been examined in active systems. Because it is chiral, ${\bar{\zeta }}_{\;pc}$ changes sign, i.e. ${\bar{\zeta }}_{\;pc}\to -{\bar{\zeta }}_{\;pc}$, when the handedness of the particles composing the system is changed. In a system that is not homochiral, its value would depend on local chiral density. Similar comments apply to all chiral terms to be discussed in this article.

The polarization equation, ignoring advection by hydrodynamic flow and motility, is (45):


(4) $$\partial_{t}P-(\Omega -\lambda A)\cdot P=-\Gamma_{P}\frac{\delta F}{\delta P},$$

where Ω and A are respectively the antisymmetric and symmetric parts of the velocity gradient tensor ∇v with components $\partial_{i}v_{j}$.

To complete the description of the polarizable and chiral active fluid, we need to specify the free energy *F*. To do this, we first separate (50) *ψ* into the small-wavenumber part $\psi_{0}$ of the active-particle concentration, and $\psi_{1}$ which is modulated on average in the columnar phase. The former plays no role in the hydrodynamics of column displacements and will not be discussed further. We then work with a free energy $F=\int_{r}[f_{P}+f_{\psi_{1},P}]$ where $f_{P}=(\alpha_{P}/2)P^{2}+(\beta_{P}/4)P^{4}+(K_{P}/2)(\nabla P{)}^{2}$ and


(5) $$\begin{array}{rl} \;f_{\psi_{1},P} & =\frac{\alpha }{2}\psi_{1}^{2}+\frac{\beta }{4}\psi_{1}^{4}+\frac{C_{∥}}{2}(\hat{P}\cdot \nabla \psi_{1}{)}^{2}\\ & \;+\frac{C_{⊥}}{2}[(I-\hat{P}\hat{P})\;:\;\nabla \nabla \psi_{1}+q_{s}^{2}\psi_{1}{]}^{2}. \end{array}$$

This concludes our construction of the model of a fluid containing chiral and polar active elements. We use this dynamical description in the next sections to obtain a theory of polar, chiral, active columnar materials in which odd elasticity emerges spontaneously.

## Breaking polar and translation symmetry: generation of odd elastic force density

While the active fluid described above contains motile units, it *cannot* form a polar nematic phase (51). This is because a uniaxial orientational order is necessarily unstable in bulk Stokesian active fluids (5, 47, 52). Instead, when $\alpha_{P}$ and *α* are *both* negative, the fluid spontaneously breaks both translation and rotation symmetries to form a polar and chiral columnar phase, at least in the absence of activity. The steady-state value of P is $P_{0}=\sqrt{|\alpha_{P}|/\beta_{P}}\hat{z}\equiv P_{0}\hat{z}$ and that of $\psi_{1}$ is:


(6) $${\bar{\psi }}_{1}=\sum_{G\in \Lambda^{*}}\psi_{1,G}e^{iG\cdot r},$$

where G are the vectors of the reciprocal lattice $\Lambda^{*}$ formed by the columnar phase in the plane transverse to $\hat{z}$. In the analysis below, we take the fundamental star of a triangular structure with the scale $|G|=q_{s}$ favored by the free energy and $\psi_{1,G}\equiv \psi_{1}^{0}=\sqrt{|\alpha |/\beta }$. Importantly, polar order evades instability (5, 47) here only because it is accompanied by *translational* order. Ignoring singularities such as dislocations (11), we now ascertain the effect of activity on the dynamics of the equilibrium columnar state.^b^ Considering broken-symmetry fluctuations about the state $\left({\bar{\psi }}_{1},P_{0}\right)$, writing the phase of ${\bar{\psi }}_{1}$ as $G\cdot (r-u_{⊥})$ and $P\approx P_{0}(\delta P_{⊥},1)$, the free energy becomes:


(7) $$\begin{array}{rl} F & =\int_{r}[\frac{\lambda }{2}(\text{Tr}\;E{)}^{2}+\mu E\;:\;E+\frac{K}{2}(\nabla^{2}u_{⊥}{)}^{2}\\ & \;+\frac{C}{2}(\delta P_{⊥}-\partial_{z}u_{⊥}{)}^{2}], \end{array}$$

where C,λ,μ,K are functions of $C_{⊥},C_{∥},\psi_{1}^{0}$ and $q_{s}$. The nonlinear elastic strain tensor E has components $E_{ij}=\frac{1}{2}(\partial_{i}u_{j}+\partial_{j}u_{i}$$-\partial_{i}u_{k}\partial_{j}u_{k}-\partial_{z}u_{i}\partial_{z}u_{j})$, with i,j,k ranging over x,y; we retain only its linearized form $E_{ij}=\frac{1}{2}(\partial_{i}u_{j}+\partial_{j}u_{i})$ in this article. We will assume, as in equilibrium systems, that $\delta P_{⊥}$ relaxes to $\partial_{z}u_{⊥}$ in a microscopic time. This assumption holds in the system under consideration provided $\zeta_{\;pa}/\eta \ll \Gamma_{P}$ (14, 47).

**Fig. 1.**
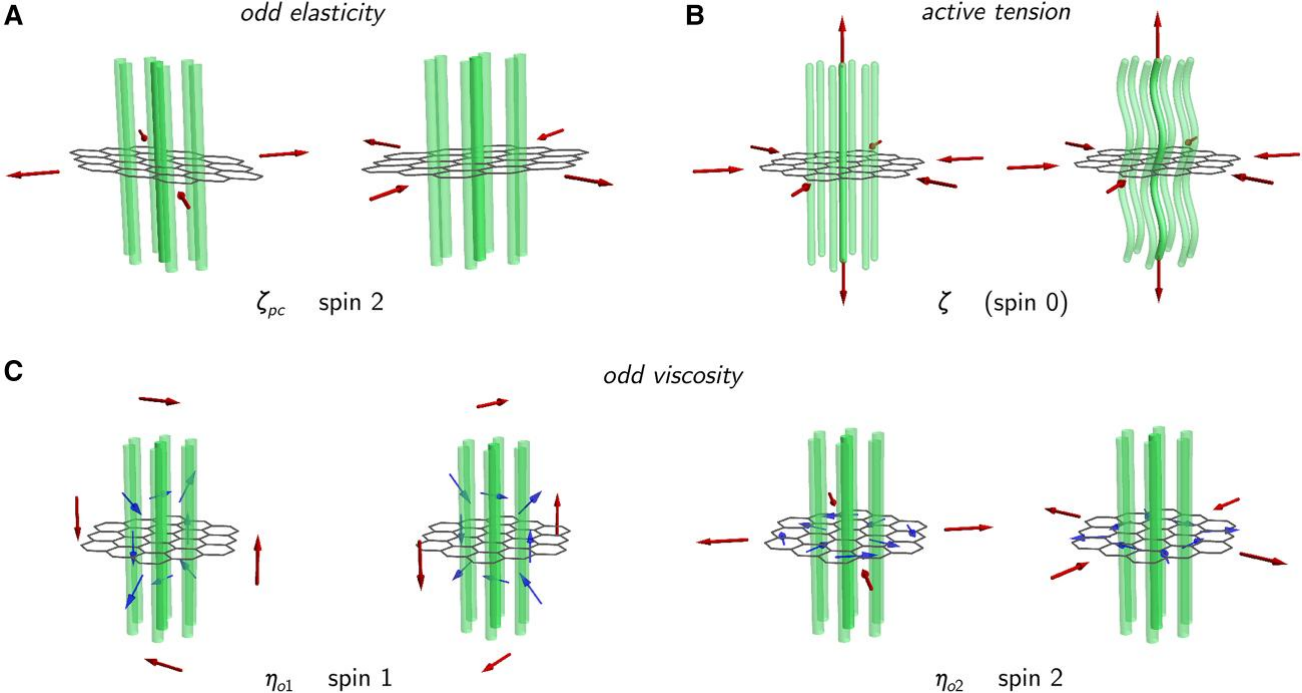
Schematic illustration of stresses (indicated by red arrows) in an active columnar phase; columns are shown as green tubes and the hexagonal arrangement by a centre-plane mesh. A) There is a single odd elastic modulus in polar chiral materials ($\zeta_{\;pc}$) coupling deviatoric elastic strains to in-plane deviatoric stresses, with a twist between polarizations; these stresses and strains carry spin 2. The elastic strains are visualized by the deformation to the regular hexagonal lattice. B) The regular dipolar activity (*ζ*) contributes an active tension and precipitates a Helfrich–Hurault-like undulational instability that is isotropic (spin 0) with respect to the elastic, in-plane directions. C) There are two odd viscous coefficients ($\eta_{o1}$ and $\eta_{o2}$) associated to twisted couplings between deviatoric shear flows in the spin 1 and spin 2 irreps and corresponding stresses. The structure of the fluid velocity in these couplings is indicated by blue arrows.

Expanding *ψ* and P in terms of $u_{⊥}$ in (2) and (3), we obtain the displacement field-dependent part of the force density whose linearized form is (Supplementary material):


(8) $$F^{e}=(\bar{\lambda }+\bar{\mu })\nabla_{⊥}\nabla_{⊥}\cdot u_{⊥}+\bar{\mu }\nabla_{⊥}^{2}u_{⊥}+\zeta \nabla^{2}u_{⊥}+\zeta_{\;pc}\nabla_{⊥}^{2}\epsilon \cdot u_{⊥},$$

where ϵ is the 2D Levi–Civita tensor and we have defined the modified Lamé coefficients $\bar{\mu }=\mu -\zeta_{1}$ and $\bar{\lambda }=\lambda -\zeta_{2}$, which are renormalized by the active terms in (3) with the *ζ*s being functions of $P_{0}$, $\psi_{1}^{0}$, $q_{s}$, $\zeta_{H}$, and $\zeta_{\;pa}$. The active force density $\zeta \nabla^{2}u_{⊥}$ has a piece $\propto \partial_{z}^{2}u_{⊥}$ (Fig. 1B) analogous to column tension, that would have been forbidden in equilibrium materials due to a combination of rotation invariance and time-reversal symmetry. This is akin to the activity-induced emergence of a layer tension in lamellar materials (10, 16, 44) or an effective elasticity modulus in active nematic elastomers (14) or a line tension or surface tension in polymers (53) or membranes (54, 55) in active fluids. The chiral force density $\propto \zeta_{\;pc}$ emerging from the ${\bar{\zeta }}_{\;pc}$ term in (3)—exactly the odd elasticity discussed in *2D* active solids (13, 15)—arises naturally in this 3D system through polarity and the spontaneous breaking of translation symmetry, (see Fig. 1A). The nondimensional ratio of odd and even shear moduli $\zeta_{\;pc}/\bar{\mu }$ was measured in a 2D active solid composed of starfish embryos (20) to be ∼7. We expect the bulk and shear moduli, $\bar{\lambda }$ and $\bar{\mu }$ to have similar magnitude; in epithelial monolayers, these are of the order of 10kPa (56). *ζ* is related to the standard active stress which is estimated to be between 5–1,000 Pa for cytoskeletal gels (57, 58) and can be higher for epithelial systems.

While the primary effect of *achiral* active force densities in the columnar materials is analogous to those in active lamellar phases—in the sense that they both endow the system with rigidities that would vanish in equilibrium—the effect of chirality in these two systems is truly distinct. In particular, the 2D odd elastic force density $\propto \zeta_{\;pc}$ has no analog in lamellar materials.

## Odd viscosity and odd elasticity

We now discuss the general form of the viscosity in a polar chiral columnar material, including its odd contributions. For a uniaxial system with at least sixfold symmetry perpendicular to the preferred axis, the deviatoric part of the velocity gradient tensor is decomposed into irreducible representations (irreps) as 1⊕2⊕2; the 1D irrep has spin 0, while the 2D irreps have spin 1 and spin 2, respectively. The viscosity tensor maps these irreps of the velocity gradients to a corresponding decomposition of the stress. The normal viscous response is an identity between corresponding irreps, implying three coefficients of viscosity^c^ in the even viscous stress: $\eta_{1}{\hat{z}}_{i}{\hat{z}}_{j}A_{zz}+2\eta A_{ij}+(\eta_{3}/2)({\hat{z}}_{i}A_{\;jz}+{\hat{z}}_{j}A_{iz})$.

Rotations within the two 2D irreps yields “odd” linear mechanical responses. Of course, a rotation requires uniquely defining a vector $w_{⊥}$ perpendicular to a given displacement or velocity w. In three dimensions, this requires chirality in the form of the Levi–Civita tensor $\epsilon_{ijk}$, a preferred axis, say the normal to the *xy* plane, and a polarity along that axis, hence a unique $\hat{z}$. We can then unambiguously write $w_{⊥i}=\epsilon_{ijz}w_{j}$. Applying this construction to the linear relation between stress and velocity gradient for any 3D chiral system with a polarity along $\hat{z}$ yields^d^ (Supplementary material):


(9) $${\sigma_{ij}}^{v}=2\eta A_{ij}+2{\left[\epsilon_{ilz}\left\{\eta_{o2}\left(A_{lj}-\delta_{\;jz}A_{lz}\right)+2\eta_{o1}\delta_{\;jz}A_{lz}\right\}\right]}^{S},$$

where $\eta_{o1},\eta_{o2}$ are the odd viscosities (59) of the spin 1 and spin 2 irreps, respectively, and we have set $\eta_{1}=\eta_{3}=0$, as these anisotropic contributions to the even viscous response do not qualitatively modify the linearized dynamics of the state of interest. We show a schematic of the two odd viscosities in Fig. 1C. The nondimensional ratios $\eta_{o1}/\eta$ and $\eta_{o2}/\eta$ are expected to increase with activity and chirality. The ratio $\eta_{o2}/\eta$ reaches as high as 1/3 in 2D chiral colloidal fluids on substrates (60, 61), which is very different from the material we consider. We see no reason why it cannot be substantially higher in 3D systems. Though we call $\eta_{o1},\eta_{o2}$ viscosities, they are not dissipative coefficients in the momentum equation: they break Onsager symmetry and have no relation to noises. Unlike the stress that ultimately resulted in 2D odd elasticity, the odd viscous stress we describe is allowed in *any* chiral material with broken time-reversal and polar anisotropy. Equation (9) implies the odd viscous force density:


(10) $$F_{o}^{v}=\eta_{o2}\nabla_{⊥}^{2}\epsilon \cdot v_{⊥}+\eta_{o1}\partial_{z}(2\Omega_{xy}\hat{z}+\partial_{z}\epsilon \cdot v_{⊥}+\epsilon \cdot \nabla_{⊥}v_{z}),$$

where the contribution $\propto \eta_{o2}$ appears in 2D, chiral active fluids (19, 30, 31) where it can be absorbed into a redefinition of the pressure in an incompressible system. In contrast, the 3D incompressibility constraint for our system permits $\eta_{o2}$ to affect bulk flows and column oscillations. The odd viscous force density $\propto \eta_{o1}$, with no analog in 2D fluids, has an important consequence for the mode structure we discuss in the next section.

We now rationalize the odd elasticity constructed in section Breaking polar and translation symmetry: generation of odd elastic force density in a manner analogous to the foregoing treatment of odd viscosities. In a columnar material, the component of strain $\partial_{z}u_{⊥}$ is absorbed by the polarization fluctuations (33) and incurs a cost only at next order in gradients in the form of column-bending elasticity. Leading-order elasticity survives only for the orthogonal strains $\nabla_{⊥}u_{⊥}$ which under the local symmetry decompose as 1⊕1⊕2. The antisymmetric part represents a rigid rotation and does not generate a stress; there are therefore *three* moduli, two even, a bulk and shear modulus, and one odd. We show a schematic of the odd elasticity in Fig. 1A. However, our approach that naturally yields odd elasticity as a consequence of translation symmetry breaking in active model H establishes it as a new generalized rigidity arising in active, chiral materials.

Less formally, there are two odd viscosities and one odd elastic modulus because the velocity field is 3D whereas the displacement field in a columnar phase has only two components. In phases with a three-component displacement field, there would be two odd elastic moduli.

## Putting everything together: wavenumber-independent odd oscillations in liquid crystals

The linearized Stokes equation containing both the odd elastic force density discussed in Sec. 2 and the odd viscous force density discussed in Sec. 3 is


(11) $$\eta \nabla^{2}v+F_{o}^{v}=\nabla \Pi -F^{e},$$

where *Π* is the pressure enforcing the 3D incompressibility constraint ∇⋅v=0. To leading order in gradients, the dynamics of the displacement field reads ${\dot{u}}_{⊥}=v_{⊥}$. Fourier transforming and solving for the velocity field yields a dynamical equation for the displacement field in the form ${\dot{u}}_{q}=M_{q}\cdot F_{q}$ (Supplementary material), where $F_{q}$ arises from $F^{e}$ and $M_{q}$ from the viscous force densities with an odd part due to $F_{o}^{v}$.

For much of the rest of the article, we will assume that the magnitudes and signs of these active terms lie in the range in which the columnar phase is linearly stable. Instabilities, when they arise, can do so through both even–even and odd–odd combinations of mobility and force density; a comment about the even–even case ζ<0 is in order here. The achiral active force density in an incompressible columnar system is *exactly* the same as that of an external stress, uniaxial along the columns or, equivalently, isotropic in the plane normal to them even considering the full nonlinear dynamics. This is analogous to active smectics (12). This implies that as in cholesterics and smectics (12), ζ<0 leads to a spontaneous Helfrich–Hurault instability (62) with, however, a degeneracy in the polarization of the 2D column-undulation field. In a finite system of lateral size *L*, this instability happens beyond a critical active stress $\pi \sqrt{K\bar{\lambda }}/L$ (Supplementary material). As in lamellar phases (12), the nonlinear mapping implies that beyond this instability an active *achiral* columnar material assumes the same state as an equilibrium columnar subject to an external stress.

We now turn to an examination of the effect of odd elasticity and viscosity on the dynamics of active, polar, and chiral columnar phases when they are linearly stable. We begin with odd elasticity alone, setting $\eta_{o1},\eta_{o2}=0$. The resulting response of the columnar material to a perturbation with wavevector $q=(q_{⊥},q_{z})=q(\sin\;\theta \cos\;\phi ,\sin\;\theta \sin\;\phi ,\cos\;\theta )$ has the form ${\dot{u}}_{q}={M_{q}}_{\mathrm{even}}\cdot ({F_{q}}_{\mathrm{odd}}+{F_{q}}_{\mathrm{even}})$. Defining its components $u_{l}=q_{⊥}\cdot u_{q}/|q_{⊥}|$ and $u_{t}=(q_{x}u_{y}-q_{y}u_{x})/|q_{⊥}|$ along and transverse to $q_{⊥}$ we find that ${F_{q}}_{\mathrm{odd}}=\zeta_{\;pc}q_{⊥}^{2}\epsilon \cdot u_{q}$, couples the $u_{l}$ and $u_{t}$ dynamics at zeroth order in wavenumber (Supplementary material):


(12) $${\dot{u}}_{l}=-\frac{(2\bar{\mu }+\bar{\lambda })q_{⊥}^{2}q_{z}^{2}+\zeta q^{2}q_{z}^{2}}{\eta q^{4}}u_{l}-\frac{\zeta_{\;pc}q_{z}^{2}q_{⊥}^{2}}{\eta q^{4}}u_{t},$$

(13) $${\dot{u}}_{t}=-\frac{\bar{\mu }q_{⊥}^{2}+\zeta q^{2}}{\eta q^{2}}u_{t}+\frac{\zeta_{\;pc}q_{⊥}^{2}}{\eta q^{2}}u_{l}.$$

**Fig. 2.**
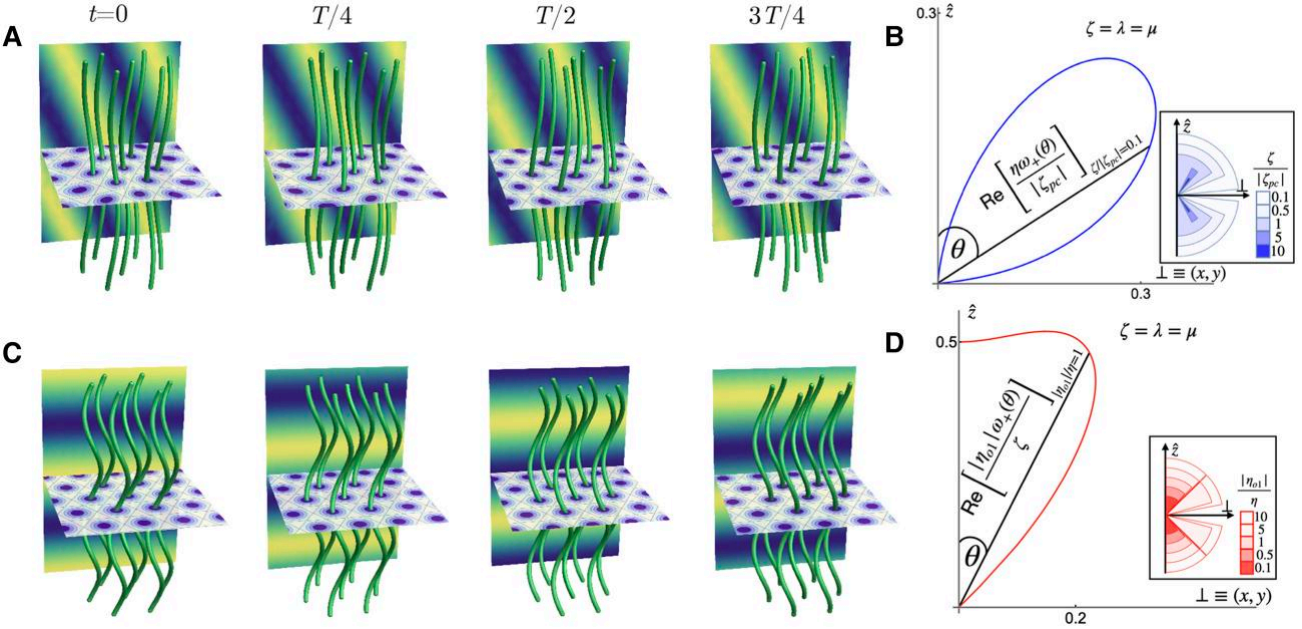
Odd dynamical oscillations in active polar chiral columnar phases. A) Odd elastic plane wave solution of (12) and (13) due to $M_{q_{\mathrm{even}}}\cdot F_{q_{\mathrm{odd}}}$; here, $\eta_{o1}=\eta_{o2}=0$ and $\zeta_{\;pc}\neq 0$. The horizontal plane shows a density plot of *ψ* and the background plane indicates the phase and direction of the plane wave. Columns are shown as green tubes and images are given for every quarter of an oscillation period (*T*). B) Polar plot of $\text{Re}[\eta \omega (\theta {)}_{+}/|\zeta_{\;pc}|]$—the oscillation frequency, nondimensionalized by the active timescale $\eta /|\zeta_{\;pc}|$—of the odd elastic oscillations due to the combination of even mobility and odd force density for λ=μ=ζ. θ=0 corresponds to a perturbation purely along the $\hat{z}$ (polarity) direction while θ=π/2 corresponds to a perturbation purely along the in-plane, crystalline directions. *Inset*: Colour-coded sectors (with different radii to ensure visibility) showing the range of *θ*—the angle between the wavevector of perturbation and $\hat{z}$—that elicits an oscillatory response for various values of $\zeta /|\zeta_{\;pc}|$. This demonstrates that the angular range increases with decreasing $\zeta /|\zeta_{\;pc}|$. While the angular range for $\zeta /|\zeta_{\;pc}|\leq 1$ extends *almost* to θ=0, there is no oscillatory response for a perturbation with wavevector along $\hat{z}$. Instead, $\text{Re}[\omega_{\pm }]\propto \theta^{2}$ at small *θ*. C) Odd oscillations from the interplay of odd mobility and even force density, $M_{q_{\mathrm{odd}}}\cdot F_{q_{\mathrm{even}}}$: plane wave solution of (15) and (16) with wavevector purely along the column axis direction. Here, $\eta_{o1}\neq 0$. D) Polar plot of $\text{Re}[|\eta_{o1}|\omega (\theta {)}_{+}/\zeta ]$—the oscillation frequency, nondimensionalized by the timescale $|\eta_{o1}|/\zeta$—of odd oscillations due to the combination of odd mobility and even force density for λ=μ=ζ. Here, $\zeta_{\;pc}=\eta_{o2}=0$. *Inset*: Colour-coded sectors (with different radii to ensure visibility) showing the range of *θ*—the angle between the wavevector of perturbation and $\hat{z}$—that elicits an oscillatory response for various values of $|\eta_{o1}|/\eta$. Note that the oscillation vanishes for θ=π/4 because the mobility due to $\eta_{o1}$ vanishes at this value in incompressible materials as can be seen from the expression of $\nu_{o}$ below (16).

This Poisson-bracket-like coupling is truly 3D, vanishing for $q_{z}=0$ or $q_{⊥}=0$.^e^ As discussed in the Introduction section, (12) and (13) lead to an oscillatory response for large-enough $\zeta_{\;pc}$, vanishing only for perturbations purely in the plane or purely along $\hat{z}$. Figure 2A shows a representative time series of the column distortions associated to this odd oscillation; the dynamics is right-handed for positive $\zeta_{\;pc}$. The general expression for the eigenfrequency displayed in Supplementary material is complicated, but its essential features can be seen in its form for θ=π/4,


(14) $$\omega_{\pm }(\theta =\pi /4)=\pm \frac{|\zeta_{\;pc}|}{2\sqrt{2}\eta }\sqrt{1-{\left(\frac{\bar{\lambda }-2\zeta }{2\sqrt{2}\zeta_{\;pc}}\right)}^{2}}-i\frac{6\zeta +4\bar{\mu }+\bar{\lambda }}{8\eta }.$$

Equation (14) describes an oscillatory mode when $|\zeta_{\;pc}|>|\bar{\lambda }-2\zeta |/2\sqrt{2}$. For a general angle θ≠0,π/2 between q and $\hat{z}$, the response is oscillatory when $\left|\zeta_{\;pc}|>|\zeta +\bar{\mu }-(2\bar{\mu }+\bar{\lambda })\cos^{2}\;\theta |/2|\cos\;\theta \right|$. This implies that when $\zeta_{\;pc}$ is larger than the other active and elastic terms, a perturbation with a wavevector *almost* fully along $\hat{z}$ also leads to an oscillatory response with Re[ω±]∝θ2 at small *θ*. We display the angular ranges in which we predict an oscillatory response in Fig. 2B for various values of $\zeta_{\;pc}$.

It is instructive to compare with the dispersive wave induced by nonreciprocity in 2D compressible solids on frictional substrates (15), where odd elasticity leads to the dynamics ${\dot{u}}_{l}\propto -q_{⊥}^{2}u_{t}$ and ${\dot{u}}_{t}\propto q_{⊥}^{2}u_{l}$. In active polar and chiral columnar phases, the presence of momentum conservation and 3D incompressibility leads to a radically different mode structure. Momentum conservation induces a long-range interaction, replacing the dispersive wave with an oscillatory mode whose frequency is nonvanishing but nonanalytic for q→0. In the presence of a momentum sink, the damping in the momentum equation would ultimately imply a dispersive wave at small wavenumbers. Indeed, if the columnar material is confined in a geometry which has a finite extent ℓ in the *z* direction, with porous walls such that the fluid can flow out of the system, then one can replace $q_{z}^{2}$ with $1/\ell^{2}$ to obtain the displacement dynamics in Ref. (15) quoted above. Note that the 3D character of the active liquid crystal remains crucial, as the mode involves a *z*-variation of the *z*-component of the velocity field at the scale of the system.

If instead the confinement is on a scale ℓ in the ⊥ plane, the odd elastic coupling for $q_{z}\ell \ll 1$ takes the form ${\dot{u}}_{l}\propto -(\zeta_{\;pc}/\eta )\ell^{2}q_{z}^{2}u_{t}$ and ${\dot{u}}_{t}\propto (\zeta_{\;pc}/\eta )u_{l}$. The possibility of active nondispersive waves à la (54, 55) is, however, ruled out by the effective damping rates (μ+ζ)/η and $(2\mu +\lambda +\zeta )\ell^{2}q_{z}^{2}/\eta$ of $u_{t}$ and $u_{l}$, respectively. The resulting eigenfrequencies $\omega_{+}=-i(\zeta +\bar{\mu })/\eta$ and $\omega_{-}=-i\ell^{2}q_{z}^{2}[(\zeta +\bar{\lambda }+2\bar{\mu })/\eta +\zeta_{\;pc}^{2}/\eta (\zeta +\bar{\mu })]$ have no real part.

We now turn to the dynamics with nonzero odd viscosities and examine their effect on the displacement dynamics focusing on the character of the oscillatory response due to the combination of ${M_{q}}_{\mathrm{odd}}$ and ${F_{q}}_{\mathrm{even}}$. The equations of motion for $u_{l}$ and $u_{t}$ are (Supplementary material):


(15) $${\dot{u}}_{l}=-\frac{\eta [(2\bar{\mu }+\bar{\lambda })q_{⊥}^{2}+\zeta q^{2}]q_{z}^{2}}{\Delta q^{4}}u_{l}+\frac{\nu_{o}(\bar{\mu }q_{⊥}^{2}+\zeta q^{2})q_{z}^{2}}{\Delta q^{4}}u_{t},$$

(16) $${\dot{u}}_{t}=-\frac{\nu_{o}[(2\bar{\mu }+\bar{\lambda })q_{⊥}^{2}+\zeta q^{2}]q_{z}^{2}}{\Delta q^{4}}u_{l}-\frac{\eta (\bar{\mu }q_{⊥}^{2}+\zeta q^{2})}{\Delta q^{2}}u_{t},$$

with $\nu_{o}=\eta_{o1}(q_{z}^{2}-q_{⊥}^{2})/q^{2}+\eta_{o2}q_{⊥}^{2}/q^{2}$ and $\Delta =\eta^{2}+\nu_{o}^{2}q_{z}^{2}/q^{2}$. We highlight a few important features of (16) and (15). As discussed in the Introduction section, odd–odd and even–even couplings of mobility and force density lead to dissipative terms, and odd–even or even–odd to reactive couplings between $u_{l}$ and $u_{t}$, as is generically the case in chiral active systems (13).

Importantly, while the odd force $\propto \zeta_{\;pc}$ vanishes for perturbations purely along the *z*-direction, the apolar and achiral active force density ∝ζ*does not*. Of the two odd contributions to the mobility, one $\propto \eta_{o2}$ vanishes for perturbations purely along $\hat{z}$, but the other $\propto \eta_{o1}$ does not. In fact, for a perturbation with θ=0 the coupled $u_{l}-u_{t}$ dynamics (15) and (16) has eigenfrequencies $\omega_{\pm }=-\zeta /(\eta \pm i\eta_{o1})$ implying an oscillatory response *even* for perturbations purely along $\hat{z}$, Fig. 2C. That is, unlike in Fig. 2B, the eigenfrequency no longer vanishes in the θ→0 limit. We emphasize that this is purely due to odd *viscosity* of 3D polar, chiral materials—and its interplay with achiral active line tension—and not odd elasticity. Significantly, a perturbation with wavevector purely in the *xy*-plane still does not elicit an oscillatory response since, due to incompressibility, ${\dot{u}}_{l}$ vanishes at this order in wavenumber. In Fig. 2D, we show a polar plot for the eigenfrequency of oscillations for arbitrary $\eta_{o1}$ and display the angular range that elicits an oscillatory response for different values of $\eta_{o1}$ in the inset. This oscillation is left-handed for positive $\eta_{o1}$.

The handedness of the oscillation for a general wavevector depends on both odd viscosities, $\eta_{o1}$ and $\eta_{o2}$, and on the direction of the mode, through the combination $\nu_{o}$. This changes sign for wavevectors making an angle $\cos^{2}\;\theta =(\eta_{o1}-\eta_{o2})/(2\eta_{o1}-\eta_{o2})$, when such a *θ* exists.

Thus, odd viscosity and odd elasticity both lead to oscillatory responses—with distinct characteristics—with wave*number*-independent frequencies thanks to the long-ranged character of Stokesian hydrodynamics. The ratio of viscosity to elasticity is a timescale, which oddness converts from the *relaxation* or *growth* time of a perturbation to the time-period of an oscillation.

## Achiral, apolar, or both

We now examine the response of higher-symmetry columnar material to perturbations, starting with systems which are neither polar nor chiral, ruling out terms that break P→−P symmetry or contain an odd number of Levi–Civita tensors. In this limit ${\bar{\zeta }}_{\;pc}$ in (3), $\zeta_{\;pc}$ in (8) and odd viscosities vanish, and


(17) $${\dot{u}}_{l}=-\frac{\cos^{2}\;\theta }{\eta }\left[(2\bar{\mu }+\bar{\lambda })\sin^{2}\;\theta +\zeta \right]u_{l},$$

(18) $${\dot{u}}_{t}=-\frac{1}{\eta }\left[\bar{\mu }\sin^{2}\;\theta +\zeta \right]u_{t},$$

are linearly decoupled. Activity, entering solely through *ζ*, still ensures that the static structure factor of displacement fluctuations, calculated by augmenting (17) and (18) with appropriate white, Gaussian noises (Supplementary material), scales as $∼1/q^{2}$ (for ζ>0) along *all* spatial directions (thereby cutting off the viscosity divergences that arise (35) in the equilibrium columnar phase^f^).

**Fig. 3.**
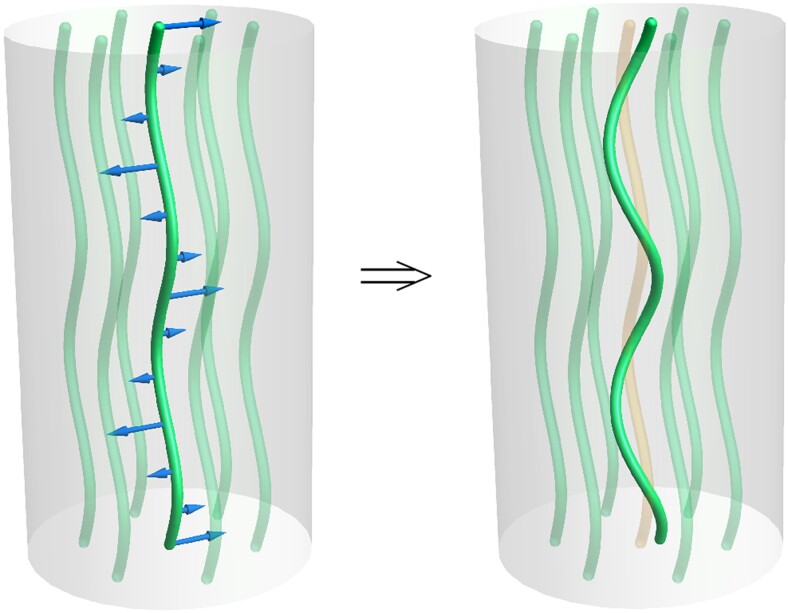
The chiral activity $\zeta_{c}$ creates transverse flows (blue arrows) to a planar column undulation (green cylinders) and resulting a helical twisting of the columns. For clarity, this is shown only for the highlighted central column, whose initial planar undulation is displayed in orange. The helices are left-handed when $\zeta_{c}$ is positive and give a macroscopic signature of the microscopic chiral activity.

We now discuss an apolar but chiral material. All the chiral effects discussed till this point also require breaking inversion symmetry. However, chiral but apolar fluids have extra active stresses which were not included in (3) because they are subdominant to the stress $\propto {\bar{\zeta }}_{\;pc}$ in polar materials. In a gradient expansion, the leading-order active chiral stress in apolar materials is $\sigma_{ij}^{ac}\propto [\epsilon_{ilk}\partial_{l}(\partial_{k}\psi \partial_{j}\psi ){]}^{S}$, introduced in Ref. (12). The resulting force density $\zeta_{c}\nabla^{2}\nabla \times u_{⊥}$ (Supplementary material) couples $u_{l}$ and $u_{t}$ fluctuations,


(19) $${\dot{u}}_{l}=-\frac{\cos^{2}\;\theta }{\eta }\left[(2\bar{\mu }+\bar{\lambda })\sin^{2}\;\theta +\zeta \right]u_{l}+\frac{iq\zeta_{c}\cos\;\theta }{\eta }u_{t},$$

(20) $${\dot{u}}_{t}=-\frac{1}{\eta }\left[\bar{\mu }\sin^{2}\;\theta +\zeta \right]u_{t}-\frac{iq\zeta_{c}\cos\;\theta }{\eta }u_{l},$$

albeit at higher gradient order than elasticity, yielding, for wavevectors purely along $\hat{z}$, mode frequencies $\omega_{\pm }=-(i/\eta )[\zeta \pm \zeta_{c}q_{z}]$. That is, chiral activity reduces the relaxation rate of one of the modes while enhancing that of the other. Another example of such a macroscopic manifestation of chirality in an active hydrodynamic instability is the preferential direction of self-shearing in epithelia (63). For $\zeta_{c}>0$ the favored mode is a left-handed helical distortion, Fig. 3. Interestingly, unlike odd elasticity in 2D chiral solids (15) and polar columnar materials discussed above, $\zeta_{c}$ here resembles a *dissipative* (though of course potentially destabilizing) Onsager coupling between $u_{t}$ and $u_{l}$. Note that this coupling originates from the same stress in active model H* that led to an odd elastic force density in active layered materials directed primarily along contours of constant mean curvature of the layers (12). For the columnar phase, in contrast, these transverse chiral flows generate a deformation of the columns into helices even at linear order (see Fig. 3).

Finally, activity fails to create a difference between apolar materials and *achiral* but polar columnar materials in the hydrodynamic limit. The lowest-order additional polar active force density $\zeta_{p}\partial_{z}\nabla^{2}u_{⊥}$ is subleading in gradients, doesn’t couple $u_{l}$ and $u_{t}$, and simply modifies $\zeta \to \zeta +i\zeta_{p}q_{z}$ in (17), (18).

## Discussion

We have described the Stokesian hydrodynamics of active columnar phases, including both chiral and polar materials. The polar chiral activity realizes an oscillatory response of the columns with a frequency that scales with wavenumber *q* as $q^{0}$ and is a ratio of either odd elasticity to even viscosity, or even elasticity to odd viscosity. Estimates for biological tissues using measurements on MDCK epithelial monolayers (56) suggest a frequency in the $10^{-3}-10^{0}$ Hz range, consistent with the timescale of odd dynamics in Ref. (20). Three-dimensionality and the viscous hydrodynamic interaction are essential to the mechanism. Active columnar phases offer an idealized representation of the odd mechanics of many living and synthetic active chiral materials. Axons and epithelia, for example, have a columnar structure and a natural polarity, so chiral activity in these tissues should generate odd dynamical effects. Columnar liquid crystals with macroscopic polarity (64), if suffused with chiral microswimmers, could realize a material in which to test our predictions. The odd dynamics of muscle tissue (65) should display distinctive contributions due to chirality.

The achiral active stress is analogous to an applied mechanical stress and produces a Helfrich–Hurault instability of the columns with degeneracy in the polarization of the undulations. In chiral active columnar materials, $\zeta_{c}$ explicitly breaks parity, lifting the degeneracy and favoring one sign of helical column undulation. This highlights the emergence of qualitatively different effects from the same chiral stress in distinct broken-symmetry phases of active model H*.

An interesting extension of our results would be to determine the effects of confinement and boundary conditions on the column undulations and their associated odd mechanics. Similarly, it will be important to determine the effect of activity on the behavior of defects (66) in columnar phases as biological tissues and filament assemblies are often highly disordered and more complex. However, the basic oscillatory dynamics we have described will still be present. Finally, our predictions are based largely on linear stability analysis. An understanding of the evolution beyond the linear regime and the final state of the system requires a direct numerical solution of the nonlinear equations of motion.

## Supplementary Material

pgae398_Supplementary_Data

## Data Availability

This article does not contain any data.

## Notes

a Here, and throughout the article, we define the spatial and temporal Fourier transform of a field a(r,t) as:

$$a_{q,\omega }=\int \frac{dt}{\sqrt{2\pi }}\int \frac{d^{3}r}{(2\pi {)}^{3/2}}e^{-i(q\cdot r-\omega t)}a(r,t).$$

b The treatment applies generically to any such state, with our ignorance of the details of the base state buried in the phenomenological coefficients (67). Departures from the generic case would require special tuning of coefficients (14, 68, 69).

c A uniaxial system has five viscosities of which the two bulk viscosities play no role in the incompressible limit.

d Odd viscosities cannot arise in 3D apolar or isotropic chiral systems (15, 70).

e Its equivalent is ruled out in an incompressible, 2D odd gel where, to $O(q^{0})$, ${\dot{u}}_{l}$ and the longitudinal velocity vanish. In the present work, however, incompressibility constrains the 3D velocity field and, therefore, ${\dot{u}}_{l}\neq 0$ at $O(q^{0})$ for a perturbation with nonzero *z* component.

f Similarly, active lamellar materials (10, 12, 14, 16, 44) escape the viscosity divergences of their equilibrium counterparts (71).

## Preprints

A preprint of this article is published at arXiv: https://arxiv.org/abs/2404.19514.
