# Supplementary material for: Chirality and odd mechanics in active columnar phases
Source: PNAS Nexus. 2024 Sep 10;3(10):pgae398. doi: 10.1093/pnasnexus/pgae398 (PMC11497608; doi:10.1093/pnasnexus/pgae398)
Supplement: pgae398_Supplementary_Data [file pgae398_supplementary_data.pdf]

Chirality and odd mechanics in active columnar phases
Supplementary Material

S. J. Kole,[1,2,3,*] Gareth P. Alexander,[4,†] Ananyo Maitra,[5,6,‡] and Sriram Ramaswamy[1,7,§]

[1]*Centre for Condensed Matter Theory, Department of Physics, Indian Institute of Science, Bangalore 560 012, India*
[2]*INI, University of Cambridge, 20 Clarkson Rd, CB3 0EH Cambridge,United Kingdom*
[3]*DAMTP, Centre for Mathematical Sciences, University of Cambridge, Wilberforce Rd, CB3 0WA Cambridge, United Kingdom*
[4]*Department of Physics, Gibbet Hill Road, University of Warwick, Coventry CV4 7AL, United Kingdom*
[5]*Laboratoire de Physique Théorique et Modélisation, CNRS UMR 8089, CY Cergy Paris Université, F-95032 Cergy-Pontoise Cedex, France*
[6]*Sorbonne Université and CNRS, Laboratoire Jean Perrin, F-75005, Paris, France*
[7]*International Centre for Theoretical Sciences, Tata Institute of Fundamental Research, Bangalore 560 089 India*

(Dated: Friday 6th September, 2024)

We provide calculational details for the results reported in the main text.

I. RECAPITULATION OF CHIRAL AND POLAR ACTIVE MODEL H

A chiral and polar active model H consists of the coupled equations describing a bulk, three-dimensional suspension of polar and chiral elements whose density is denoted by $\psi(\mathbf{r},t)$, velocity $\mathbf{v}(\mathbf{r},t)$ and the degree of polar orientation is accounted for by the polar order parameter $\mathbf{P}(\mathbf{r},t)$, where $\mathbf{r}=(x,y,z)$. The dynamical equation for a conserved scalar field ψ is given by

$$\partial_t\psi = -\nabla\cdot(\psi\mathbf{v}) + M\nabla^2\frac{\delta F}{\delta\psi} + \nabla\cdot(\psi\mathbf{V}_a) + \xi_\psi, \tag{1}$$

where

$$\mathbf{V}_a = \kappa_1(\nabla\psi)\nabla^2\psi + \kappa_2\nabla(\psi\nabla^2\psi) + v_p\mathbf{P}, \tag{2}$$

accounts for the active current ignored in the main text (because they turn out to not affect the hydrodynamics of the columnar phase) and $\langle\xi_\psi(\mathbf{r},t)\xi_\psi(\mathbf{r}',t')\rangle = -2D\nabla^2\delta(\mathbf{r}-\mathbf{r}')\delta(t-t')$ is a conserving noise also ignored in the main text, which only discussed the deterministic dynamics. The free energy F will be discussed in the next sections. In principle, the mobility M is a tensor that depends on $\mathbf{P}$; $M_{ij} = m_1\delta_{ij} + m_2P_iP_j + \ldots$. Such an anisotropic mobility implies that the dissipative passive term in the ψ equation generally has the form $M_{ij}\nabla_i\nabla_j(\delta F/\delta\psi)$. Similarly, the noise covariance should be $-2D_{ij}\nabla_i\nabla_j\delta(\mathbf{r}-\mathbf{r}')\delta(t-t')$, with $D_{ij} = d_1\delta_{ij} + d_2P_iP_j + \ldots$. However, we ignore the tensorial character of the mobility or the noise covariance here. The mobility only affects the permeation of the displacement fields in the columnar phase we are interested in, and its dependence on $\mathbf{P}$ makes the permeation coefficient anisotropic. However, in momentum-conserved systems that we will consider, permeation yields a sub-leading correction to the leading order in wavenumber behaviour. In fact, we will have no occasion to consider permeation at all. The velocity field, in the limit of a small Reynolds number relevant for most biological and soft matter systems, has an overdamped, Stokesian dynamics:

$$\eta_{ijkl}\partial_j\partial_k v_l = \psi\partial_i\frac{\delta F[\psi]}{\delta\psi} + \partial_i\Pi - \partial_j\sigma^a_{ij} + \xi_{v_i}, \tag{3}$$

where Π is the pressure that enforces the incompressibility constraint $\nabla\cdot\mathbf{v}=0$, we will consider only one even viscosity in η_{ijkl} and $\langle\xi_{v_i}(\mathbf{r},t)\xi_{v_j}(\mathbf{r}',t')\rangle = -2\delta_{ij}D_v\nabla^2\delta(\mathbf{r}-\mathbf{r}')\delta(t-t')$. The active stress $\boldsymbol{\sigma}^a$ and the viscosity tensor $\boldsymbol{\eta}$ will be discussed in the next sections. In equilibrium, the noise strengths are related via $D/M = D_v/\eta$, where η is the even viscous coefficient. Finally, the equation of motion for the polar order parameter is

$$\partial_t P_i + (\lambda_P P_j + v_j)\partial_j P_i - (\Omega_{ij} - \lambda A_{ij})P_j = -\Gamma_P\frac{\delta F}{\delta P_i} + \xi_{P_i}, \tag{4}$$

where advective and self-advective terms which will turn out to be irrelevant for the dynamics of the columnar phase have been explicitly retained. Here, $\Omega_{ij} = (1/2)(\partial_j v_i - \partial_i v_j)$ and $A_{ij} = (1/2)(\partial_j v_i + \partial_i v_j)$. ξ_{P_i} is a Gaussian white noise with a variance D_P. Again, here Γ_P can, in principle, be anisotropic and depend on $\mathbf{P}$ itself. However, this anisotropy has no influence on the hydrodynamic behaviour we are interested in, and we therefore do not display it.

II. COLUMNAR DENSITY WAVE AND ACTIVE STRESSES

We describe the columnar order by the spatially modulated part ψ_1 of a number density field coupled to a polar order parameter $\mathbf{P}$ that orients the columns with an equilibrium free energy

$$F = \int \left\{ \frac{\alpha}{2}\psi_1^2 + \frac{\beta}{4}\psi_1^4 + \frac{C_\parallel}{2}\left(\hat{\mathbf{P}}\cdot\nabla\psi_1\right)^2 + \frac{C_\perp}{2}\left[(\mathbf{I}-\hat{\mathbf{P}}\hat{\mathbf{P}}):\nabla\nabla\psi_1 + q_s^2\psi_1\right]^2 + \frac{\alpha_P}{2}|\mathbf{P}|^2 + \frac{\beta_P}{4}|\mathbf{P}|^4 + \frac{K_P}{2}\left|\nabla\mathbf{P}\right|^2 \right\} dV. \tag{5}$$

The couplings between $\mathbf{P}$ and gradients of ψ suppress gradients parallel to $\mathbf{P}$ and produce Brazovskii-like ordering at wavenumber q_s in directions orthogonal to $\mathbf{P}$; the operator $\mathbf{I}-\hat{\mathbf{P}}\hat{\mathbf{P}}$ is the projector onto the plane orthogonal to $\mathbf{P}$. A free energy of the same structure also describes an apolar columnar phase with the liquid crystal director replacing the polar order parameter $\mathbf{P}$. Polar columnar ground states are obtained when both $\alpha_P < 0$ and $\alpha < 0$ with a uniform polar order parameter $\hat{\mathbf{P}} = \hat{\mathbf{z}}$ and a spatially-modulated density field

$$\bar{\psi}_1 = \sum_{\mathbf{G}\in\Lambda^*} \psi_{1,\mathbf{G}}\, \mathrm{e}^{i\mathbf{G}\cdot\mathbf{x}}, \tag{6}$$

where Λ^* is a reciprocal lattice in the xy-plane orthogonal to $\mathbf{P}$. We consider only hexagonal lattices and limit the Fourier series to the fundamental star, explicitly

$$\mathbf{G} \in \left\{ \pm q_s\hat{\mathbf{x}}, \pm q_s\,\frac{-\hat{\mathbf{x}}+\sqrt{3}\,\hat{\mathbf{y}}}{2}, \pm q_s\,\frac{\hat{\mathbf{x}}+\sqrt{3}\,\hat{\mathbf{y}}}{2} \right\}. \tag{7}$$

By symmetry, the amplitudes $\psi_{1,\mathbf{G}}$ all have the same magnitude and we take them to be real, $\psi_{1,\mathbf{G}} = \psi_1^0$ for all $\mathbf{G}$.

Hydrodynamic fluctuations of the columnar state are obtained by introducing an Eulerian displacement field $\mathbf{u}_\perp$, having components only in the xy-plane orthogonal to $\hat{\mathbf{z}}$, into the density modulation

$$\psi_1 = \sum_{\mathbf{G}} \psi_0\, \mathrm{e}^{i\mathbf{G}\cdot(\mathbf{x}-\mathbf{u}_\perp)}, \tag{8}$$

coupled with variations in the direction of polar alignment, $\hat{\mathbf{P}} = \hat{\mathbf{z}} + \delta\mathbf{P}_\perp$. The free energy (5) then reproduces the usual elasticity of columnar phases [1] which reads

$$F_{\mathbf{u}_\perp,\delta\mathbf{P}_\perp} = \int \frac{1}{2}\left[\lambda \mathrm{Tr}[\mathsf{E}]^2 + 2\mu\mathsf{E}:\mathsf{E} + K\nabla^2 u_k\nabla^2 u_k + C(\delta\mathbf{P}_\perp - \partial_z\mathbf{u}_\perp)^2\right] dV \tag{9}$$

where $C = 3C_\parallel {\psi_1^0}^2 q_s^2$, $\lambda = 3C_\perp {\psi_1^0}^2 q_s^4$, $\mu = 3C_\perp {\psi_1^0}^2 q_s^4$, $K = 3C_\perp {\psi_1^0}^2 q_s^2$ and we have defined a rotation-invariant strain tensor

$$\begin{aligned} E_{ij} &= \frac{1}{2}[\partial_i u_j + \partial_j u_i - \partial_i u_k \partial_j u_k - \partial_z u_i \partial_z u_j] \\ &= \frac{1}{2}\begin{pmatrix} 2\partial_x u_x - (\partial_x u_x)^2 - (\partial_x u_y)^2 - (\partial_z u_x)^2 & \partial_x u_y + \partial_y u_x - \partial_x u_x \partial_y u_x - \partial_x u_y \partial_y u_y - \partial_z u_x \partial_z u_y \\ \partial_x u_y + \partial_y u_x - \partial_x u_x \partial_y u_x - \partial_x u_y \partial_y u_y - \partial_z u_x \partial_z u_y & 2\partial_y u_y - (\partial_y u_x)^2 - (\partial_y u_y)^2 - (\partial_z u_y)^2 \end{pmatrix}. \end{aligned} \tag{10}$$

The suppression of ψ gradients parallel to $\mathbf{P}$ leads to a coupling $\propto |\delta\mathbf{P}_\perp - \partial_z\mathbf{u}_\perp|^2$, which expresses that pure tilts of the columns do not elicit elastic stresses at linear order. This implies that polarisation fluctuations relax to those determined by the displacement field in a microscopic time (at least for small activities). Since the polarisation fluctuations are not hydrodynamic and relax fast to values determined by displacement fluctuations, the advective and self-advective terms in (4) yield terms at higher order in gradients than we retain and are irrelevant for the hydrodynamics of the polar columnar phase.

The dynamics of the Eulerian displacement field is obtained from (1)

$$\partial_t\mathbf{u}_\perp = \mathbf{v}_\perp - \mathbf{v}\cdot\nabla\mathbf{u}_\perp - \Gamma\frac{\delta F_{\mathbf{u}}}{\delta\mathbf{u}_\perp} + \mathbf{V}_{a\perp} + \boldsymbol{\xi}_u, \tag{11}$$

where $\boldsymbol{\xi}_u$ is a white, Gaussian noise, $\Gamma \propto Mq_s^2$ and $\mathbf{V}_{a\perp}$ is the active permeation which, for simplicity, we only write to linear order:

$$\mathbf{V}_{a\perp} = \mu_1\nabla^2\mathbf{u}_\perp + \mu_2\nabla_\perp(\nabla_\perp\cdot\mathbf{u}_\perp), \tag{12}$$

where μ_1 and μ_2 depend on $\kappa_{1,2}$ and v_p. However, these terms at $\mathcal{O}(\nabla^2)$ are subdominant to the terms that appear through $\mathbf{v}_\perp$ due to Stokesian dynamics, and therefore, we do not consider them further.

We focus now on the hydrodynamic form of the active contributions to the stress. We consider four distinct active stresses: an apolar achiral stress proportional to $\nabla\psi\nabla\psi$; a similar term proportional to $\mathbf{PP}$; a polar chiral stress proportional to the symmetric part of $(\mathbf{P}\times\nabla\psi)\,\nabla\psi$; and an apolar chiral stress proportional to the symmetric part of $\nabla\times(\nabla\psi\nabla\psi)$ (which we discuss later). We treat the term proportional to $\mathbf{PP}$ first: its linearisation is

$$\mathbf{PP} = P_0^2\big(\hat{\mathbf{z}}\hat{\mathbf{z}} + \hat{\mathbf{z}}\,\delta\mathbf{P}_\perp + \delta\mathbf{P}_\perp\,\hat{\mathbf{z}}\big) = P_0^2\big(\hat{\mathbf{z}}\hat{\mathbf{z}} + \hat{\mathbf{z}}\,\partial_z\mathbf{u}_\perp + \partial_z\mathbf{u}_\perp\,\hat{\mathbf{z}}\big), \tag{13}$$

where $P_0 = \sqrt{-\alpha_p/\beta_P}$ is the preferred magnitude of $\mathbf{P}$. The analysis of the stresses involving ψ all follow from the hydrodynamic part of $\nabla\psi\nabla\psi$, which is

$$\nabla\psi\nabla\psi = \sum_{\mathbf{G}_1,\mathbf{G}_2}\psi_0^2\big[(\mathbf{I}-\nabla\mathbf{u}_\perp)\cdot i\mathbf{G}_1\big]\big[(\mathbf{I}-\nabla\mathbf{u}_\perp)\cdot i\mathbf{G}_2\big]\,\mathrm{e}^{i(\mathbf{G}_1+\mathbf{G}_2)\cdot(\mathbf{x}-\mathbf{u}_\perp)}, \tag{14}$$

$$= \sum_{\mathbf{G}}\psi_0^2\big(\mathbf{I}-\nabla\mathbf{u}_\perp\big)\cdot\mathbf{GG}\cdot\big(\mathbf{I}-(\nabla\mathbf{u}_\perp)^T\big) + \cdots, \tag{15}$$

where we retain explicitly only the terms with $\mathbf{G}_1+\mathbf{G}_2 = 0$. For the fundamental star of the hexagonal lattice

$$\sum_{\mathbf{G}}\mathbf{GG} = 3q_s^2\big(\hat{\mathbf{x}}\hat{\mathbf{x}} + \hat{\mathbf{y}}\hat{\mathbf{y}}\big), \tag{16}$$

so that to linear order (and retaining only the hydrodynamic part)

$$\nabla\psi\nabla\psi = 3q_s^2\psi_0^2\big(\hat{\mathbf{x}}\hat{\mathbf{x}} + \hat{\mathbf{y}}\hat{\mathbf{y}} - \nabla\mathbf{u}_\perp - (\nabla\mathbf{u}_\perp)^T\big). \tag{17}$$

The full, nonlinear expression for $\nabla\psi\nabla\psi$ is

$$\nabla\psi\nabla\psi = 3{\psi_1^0}^2 q_s^2\begin{pmatrix} (\partial_x u_x - 1)^2 + (\partial_x u_y)^2 & \partial_y u_x(\partial_x u_x - 1) + (\partial_y u_y - 1)\partial_x u_y & \partial_z u_x(\partial_x u_x - 1) + \partial_z u_y \partial_x u_y \\ \partial_y u_x(\partial_x u_x - 1) + \partial_x u_y(\partial_y u_y - 1) & (\partial_y u_x)^2 + (\partial_y u_y - 1)^2 & \partial_z u_x \partial_y u_x + \partial_z u_y(\partial_y u_y - 1) \\ \partial_z u_x(\partial_x u_x - 1) + \partial_z u_y \partial_x u_y & \partial_z u_x \partial_y u_x + \partial_z u_y(\partial_y u_y - 1) & (\partial_z u_x)^2 + (\partial_z u_y)^2 \end{pmatrix}. \tag{18}$$

For the polar chiral contribution a direct calculation gives

$$\begin{aligned}\big(\mathbf{P}\times\nabla\psi\big)\nabla\psi = 3q_s^2 P_0\psi_0^2\Big[&\hat{\mathbf{y}}\hat{\mathbf{x}} - \hat{\mathbf{x}}\hat{\mathbf{y}} + \big(\partial_x u_y + \partial_y u_x\big)\big(\hat{\mathbf{x}}\hat{\mathbf{x}} - \hat{\mathbf{y}}\hat{\mathbf{y}}\big) + \big(\partial_y u_y - \partial_x u_x\big)\big(\hat{\mathbf{x}}\hat{\mathbf{y}} + \hat{\mathbf{y}}\hat{\mathbf{x}}\big) \\ &+ \big(\partial_x u_x + \partial_y u_y\big)\big(\hat{\mathbf{x}}\hat{\mathbf{y}} - \hat{\mathbf{y}}\hat{\mathbf{x}}\big) + \partial_z u_x\big(\hat{\mathbf{z}}\hat{\mathbf{y}} - \hat{\mathbf{y}}\hat{\mathbf{z}}\big) + \partial_z u_y\big(\hat{\mathbf{x}}\hat{\mathbf{z}} - \hat{\mathbf{z}}\hat{\mathbf{x}}\big)\Big],\end{aligned} \tag{19}$$

retaining only the linear hydrodynamic terms.

Combining these results we see that the active stress

$$\boldsymbol{\sigma}^a = -\zeta_H\nabla\psi\nabla\psi + \zeta_{pa}\,\mathbf{PP} + \bar{\zeta}_{pc}\big[(\mathbf{P}\times\nabla\psi)\nabla\psi\big]^S, \tag{20}$$

where $\mathbf{M}^S$ denotes the symmetrisation of the tensor $\mathbf{M}$, implies a hydrodynamic active force density

$$\nabla\cdot\boldsymbol{\sigma}^a = 3q_s^2\psi_0^2\zeta_H\big(\nabla^2\mathbf{u} + \nabla(\nabla_\perp\cdot\mathbf{u}_\perp)\big) + \zeta_{pa}P_0^2\big(\partial_z^2\mathbf{u}_\perp + \hat{\mathbf{z}}\,\partial_z(\nabla_\perp\cdot\mathbf{u}_\perp)\big) + 3q_s^2P_0\psi_0^2\bar{\zeta}_{pc}\nabla_\perp^2\big(u_y\,\hat{\mathbf{x}} - u_x\,\hat{\mathbf{y}}\big). \tag{21}$$

This can be rewritten as

$$\nabla\cdot\boldsymbol{\sigma}^a = (3q_s^2\psi_0^2\zeta_H + \zeta_{pa}P_0^2)\nabla^2\mathbf{u} - \zeta_{pa}P_0^2\big[\nabla_\perp^2\mathbf{u}_\perp + \nabla_\perp(\nabla_\perp\cdot\mathbf{u}_\perp)\big] + 3q_s^2P_0\psi_0^2\bar{\zeta}_{pc}\nabla_\perp^2\big(u_y\,\hat{\mathbf{x}} - u_x\,\hat{\mathbf{y}}\big). \tag{22}$$

upon absorbing total gradients arising from ζ_H and ζ_{pa} into a redefinition of the pressure. After combining with the passive elasticity, this gives the elastic force density $\boldsymbol{\mathcal{F}}^e$ of Eq. (8) in the main text with the definitions $\bar{\mu} = \mu - \zeta_1$ and $\bar{\lambda} = \lambda - \zeta_2$ and $\zeta_1 = \zeta_{pa}P_0^2$, $\zeta_2 = 0$, $\zeta = (3q_s^2\psi_0^2\zeta_H + \zeta_{pa}P_0^2)$ and $\zeta_{pc} = 3q_s^2P_0\psi_0^2\bar{\zeta}_{pc}$. We retain ζ_2 (which is 0 in our description) to remind the reader that, in general, the bulk modulus is renormalised by activity (for instance, if one retains an anisotropic version of the $\nabla\psi\nabla\psi$ stress).

While in this article, we confine ourselves to a discussion of the linear physics of active columnar materials, note that our description that starts from an active model H automatically yields the fully covariant nonlinear equations of motion for the active columnar phase (as we show here) which should serve as the starting point for a numerical simulation of such materials.

III. ODD VISCOSITY IN POLAR AND CHIRAL FLUIDS

In equilibrium polar fluids, with at least six-fold symmetry transverse to the polar direction, the viscosity tensor has the following form:

$$\eta_{ijkl} = \eta_1 P_i P_j P_k P_l + \eta(\delta_{ik}\delta_{jl} + \delta_{il}\delta_{jk}) + \frac{\eta_3}{4}(P_i P_k \delta_{jl} + P_j P_k \delta_{il} + P_i P_l \delta_{jk} + P_j P_l \delta_{ik}) + \eta_4 \delta_{ij}\delta_{kl} + \eta_5(\delta_{ij} P_k P_l + P_i P_j \delta_{kl}). \quad (23)$$

The last two vanish in incompressible systems leaving a viscous stress tensor

$$\sigma_{ij}{}_u^v = \eta_1 P_i P_j P_k P_l A_{kl} + 2\eta A_{ij} + \frac{\eta_3}{2}(P_i P_k A_{jk} + P_j P_k A_{ik}). \quad (24)$$

Of these, we only retain η in our discussion in the main text and will do so here as well. In chiral and polar materials, one has additional velocity-dependent active stresses at the same order in gradients

$$\sigma_{ij}{}_o^v = 2\left[\epsilon_{ilk} P_k \left\{2\eta_{o1} P_j P_m A_{lm} + \eta_{o2}\left(A_{lj} - P_j P_m A_{lm}\right)\right\}\right]^S . \quad (25)$$

The divergence of the viscous stresses $\partial_j(\sigma_{ij}{}_o^v + \sigma_{ij}{}_u^v) = \eta_{ijkl}\partial_j\partial_k v_l$ gives the L.H.S. of (3) (with $\eta_1 = \eta_3 = 0$). In particular, the linearised version of (25)

$$\boldsymbol{\mathcal{F}}_o^v = \eta_{o1}\big(\partial_{zz}\boldsymbol{\epsilon}\cdot\mathbf{v}_\perp + \boldsymbol{\epsilon}\cdot\nabla_\perp\partial_z v_z + \partial_z(\partial_x v_y - \partial_y v_x)\,\hat{\mathbf{z}}\big) + \eta_{o2}\nabla_\perp^2\boldsymbol{\epsilon}\cdot\mathbf{v}_\perp, \quad (26)$$

is the odd viscous force density (Eq. (10) of the main text).

IV. LINEAR HYDRODYNAMICS OF ACTIVE COLUMNAR PHASES

The hydrodynamic variables in the columnar phase are the Eulerian displacement field $\mathbf{u}_\perp$ of the columns and the fluid velocity $\mathbf{v}$. Neglecting permeation, the linear hydrodynamics of the displacement field is

$$\partial_t \mathbf{u}_\perp = \mathbf{v}_\perp, \quad (27)$$

while that for the fluid velocity is the Stokes equation (Eq. (11) of the main text)

$$0 = -\nabla\Pi + \eta\nabla^2\mathbf{v} + \boldsymbol{\mathcal{F}}_o^v + \boldsymbol{\mathcal{F}}^e, \quad (28)$$

together with incompressibility $\nabla\cdot\mathbf{v} = 0$. Here, Π is the pressure and $\boldsymbol{\mathcal{F}}^e$ is the elastic force density (Eq. (8) of the main text)

$$\boldsymbol{\mathcal{F}}^e = \bar{\mu}\nabla_\perp^2\mathbf{u}_\perp + (\bar{\mu} + \bar{\lambda})\nabla_\perp\nabla_\perp\cdot\mathbf{u}_\perp + \zeta\nabla^2\mathbf{u}_\perp + \zeta_{pc}\nabla_\perp^2\boldsymbol{\epsilon}\cdot\mathbf{u}_\perp. \quad (29)$$

We solve the Stokes equation for the velocity in terms of the displacement field by Fourier transform

$$0 = -i\,\mathbf{q}\,\tilde{\Pi} - \eta q^2\,\tilde{\mathbf{v}} - \eta_{o1}\Big(q_z^2\,\boldsymbol{\epsilon} + q_z\big[(\boldsymbol{\epsilon}\cdot\mathbf{q}_\perp)\hat{\mathbf{z}} - \hat{\mathbf{z}}(\boldsymbol{\epsilon}\cdot\mathbf{q}_\perp)\big]\Big)\cdot\tilde{\mathbf{v}} - \eta_{o2}q_\perp^2\,\boldsymbol{\epsilon}\cdot\tilde{\mathbf{v}}_\perp + \mathbf{F}_{\mathbf{q}} \quad (30)$$

In developing the solution we make use of the natural decomposition of the vector space $\mathbb{R}^3$ as $\mathbb{R}^2 \oplus \mathbb{R}$. There are two such splittings: One comes from the columnar structure, where the (polar) orientation of the columns defines the $\mathbb{R}$ factor, which we take to be $\hat{\mathbf{z}}$, and the vector $\tilde{\mathbf{u}}_\perp$ lies entirely in the two-dimensional subspace orthogonal to this (xy-plane). The other comes from the wavevector $\mathbf{q}$, whose direction also provides a splitting $\mathbb{R}^3 \cong \mathbb{R}^2 \oplus \mathbb{R}$, and as the flow is incompressible, $\mathbf{q}\cdot\tilde{\mathbf{v}} = 0$, the velocity $\tilde{\mathbf{v}}$ lies entirely in the orthogonal two-dimensional subspace. Since our objective here is to solve for $\tilde{\mathbf{v}}$ as a function of $\tilde{\mathbf{u}}$, we make use of the latter splitting.

Let $\{\mathbf{e}_1, \mathbf{e}_2, \mathbf{e}_3\}$ be an orthonormal frame with $\mathbf{q} = q\,\mathbf{e}_3$ and $\hat{\mathbf{z}}$ lying in the $\mathbf{e}_1, \mathbf{e}_3$-plane (*i.e.* $\mathbf{e}_2$ directs the intersection of the plane orthogonal to $\mathbf{q}$ with the xy-plane). This amounts to

$$\hat{\mathbf{z}} = \frac{q_z}{q}\,\mathbf{e}_3 - \frac{q_\perp}{q}\,\mathbf{e}_1, \qquad \mathbf{q}_\perp = \frac{q_\perp^2}{q}\,\mathbf{e}_3 + \frac{q_z q_\perp}{q}\,\mathbf{e}_1, \qquad \boldsymbol{\epsilon}\cdot\mathbf{q}_\perp = -q_\perp\,\mathbf{e}_2. \quad (31)$$

In this basis the Stokes equation reads

$$\begin{aligned} 0 = &-\left[\eta q^2\big(\mathbf{e}_1\mathbf{e}_1 + \mathbf{e}_2\mathbf{e}_2\big) - \big[\eta_{o1}(q_z^2 - q_\perp^2) + \eta_{o2}q_\perp^2\big]\frac{q_z}{q}\big(\mathbf{e}_2\mathbf{e}_1 - \mathbf{e}_1\mathbf{e}_2\big)\right]\cdot\tilde{\mathbf{v}} + \big(\mathbf{e}_1\mathbf{e}_1 + \mathbf{e}_2\mathbf{e}_2\big)\cdot\mathbf{F}_{\mathbf{q}} \\ &+ \mathbf{e}_3\left[-iq\,\tilde{\Pi} - \frac{(2\eta_{o1}q_z^2 + \eta_{o2}q_\perp^2)q_\perp}{q}\,\mathbf{e}_2\cdot\tilde{\mathbf{v}} + \mathbf{e}_3\cdot\mathbf{F}_{\mathbf{q}}\right], \end{aligned} \quad (32)$$

The component parallel to $\mathbf{q}$ gives an expression for the pressure

$$i\tilde{\Pi} = -\frac{(2\eta_{o1}q_z^2 + \eta_{o2}q_\perp^2)q_\perp}{q^2}\,\mathbf{e}_2 \cdot \tilde{\mathbf{v}} + \frac{1}{q}\,\mathbf{e}_3 \cdot \mathbf{F}_\mathbf{q}, \tag{33}$$

while the orthogonal components give the fluid velocity as

$$\tilde{\mathbf{v}} = \left[\frac{\eta}{\Delta q^2}\big(\mathbf{e}_1\mathbf{e}_1 + \mathbf{e}_2\mathbf{e}_2\big) + \frac{\nu_o q_z}{\Delta q^3}\big(\mathbf{e}_2\mathbf{e}_1 - \mathbf{e}_1\mathbf{e}_2\big)\right] \cdot \mathbf{F}_\mathbf{q}, \tag{34}$$

where

$$\nu_o = \frac{\eta_{o1}(q_z^2 - q_\perp^2) + \eta_{o2}q_\perp^2}{q^2}, \tag{35}$$

$$\Delta = \eta^2 + \frac{\nu_o^2 q_z^2}{q^2}. \tag{36}$$

The two terms in the inverse viscosity operator are the even and odd parts of the mobility $\mathbf{M}_\mathbf{q}$. It can be seen directly that the odd mobility vanishes if $q_z = 0$. It also vanishes along the cone $\nu_o = 0$, or

$$\frac{q_z^2}{q^2} = \frac{\eta_{o1} - \eta_{o2}}{2\eta_{o1} - \eta_{o2}}, \tag{37}$$

and changes sign between the inside and outside of this cone. The consequence of this is that the sense of rotation in the column oscillations can switch as a function of the direction of the wave relative to the column axis.

V. DISPLACEMENT DYNAMICS

The dynamics of the displacement field, $\partial_t \tilde{\mathbf{u}}_\perp = \tilde{\mathbf{v}}_\perp$, takes place in the xy-plane orthogonal to the (polar) column axis and it is convenient to express it using a basis adapted to this splitting. Let $\{\mathbf{e}_l, \mathbf{e}_t, \hat{\mathbf{z}}\}$ be an orthonormal frame with $\mathbf{q} = q_z\,\hat{\mathbf{z}} + q_\perp\,\mathbf{e}_l$. One finds

$$\mathbf{e}_1 = \frac{q_z}{q}\,\mathbf{e}_l - \frac{q_\perp}{q}\,\hat{\mathbf{z}}, \qquad\qquad \mathbf{e}_2 = \mathbf{e}_t, \tag{38}$$

and then the displacement dynamics reads

$$\partial_t \tilde{\mathbf{u}}_\perp = \left[\frac{\eta}{\Delta q^2}\left(\frac{q_z^2}{q^2}\mathbf{e}_l\mathbf{e}_l + \mathbf{e}_t\mathbf{e}_t\right) - \frac{\nu_o q_z^2}{\Delta q^4}\,\boldsymbol{\epsilon}\right] \cdot \mathbf{F}_\mathbf{q} \tag{39}$$

In the same basis the elastic force density is

$$\mathbf{F}_\mathbf{q} = -\left[\big(\bar{\mu}q_\perp^2 + \zeta q^2\big)\big(\mathbf{e}_l\mathbf{e}_l + \mathbf{e}_t\mathbf{e}_t\big) + (\bar{\mu} + \bar{\lambda})q_\perp^2\,\mathbf{e}_l\mathbf{e}_l + \zeta_{pc}q_\perp^2\,\boldsymbol{\epsilon}\right] \cdot \tilde{\mathbf{u}}_\perp. \tag{40}$$

The structure of the linear dynamics is $\partial_t \tilde{\mathbf{u}}_\perp = \mathbf{D} \cdot \tilde{\mathbf{u}}_\perp$, where the 'dynamical matrix' $\mathbf{D}$ can be written (in the $\mathbf{e}_l, \mathbf{e}_t$ basis) in the form

$$\mathbf{D} = D_0 \begin{bmatrix} 1 & 0 \\ 0 & 1 \end{bmatrix} + D_\mathrm{r} \begin{bmatrix} 0 & -1 \\ 1 & 0 \end{bmatrix} + D_+ \begin{bmatrix} 1 & 0 \\ 0 & -1 \end{bmatrix} + D_\times \begin{bmatrix} 0 & 1 \\ 1 & 0 \end{bmatrix}, \tag{41}$$

and the coefficients are

$$D_0 = -\frac{\eta}{2\Delta q^4}\left[\big(\bar{\mu}q_\perp^2 + \zeta q^2\big)\big(2q_z^2 + q_\perp^2\big) + (\bar{\mu} + \bar{\lambda})q_z^2 q_\perp^2\right] - \frac{\nu_o \zeta_{pc} q_z^2 q_\perp^2}{\Delta q^4}, \tag{42}$$

$$D_\mathrm{r} = \frac{\eta\zeta_{pc}(2q_z^2 + q_\perp^2)q_\perp^2}{2\Delta q^4} - \frac{\nu_o q_z^2}{2\Delta q^4}\big[2\big(\bar{\mu}q_\perp^2 + \zeta q^2\big) + (\bar{\mu} + \bar{\lambda})q_\perp^2\big], \tag{43}$$

$$D_+ = \frac{\eta q_\perp^2}{2\Delta q^4}\big[\bar{\mu}q_\perp^2 + \zeta q^2 - (\bar{\mu} + \bar{\lambda})q_z^2\big], \tag{44}$$

$$D_\times = \frac{\eta\zeta_{pc}q_\perp^4}{2\Delta q^4} - \frac{\nu_o(\bar{\mu} + \bar{\lambda})q_z^2 q_\perp^2}{2\Delta q^4}. \tag{45}$$

The character of the dynamics depends on the sign of $D_{\rm r}^2 - D_+^2 - D_\times^2$; when it is positive the dynamics is oscillatory, which is the regime we focus on. The eigenmodes in the oscillatory regime are given by

$$\tilde{\mathbf{u}} = u_0 \big[(D_{\rm r} - D_\times)\, \mathbf{e}_l + (D_+ \pm i\omega)\, \mathbf{e}_t \big]\, \mathrm{e}^{D_0 t \mp i\omega t}, \tag{46}$$

where $\omega = \sqrt{D_{\rm r}^2 - D_+^2 - D_\times^2}$ is the oscillation frequency. Growth or decay of these modes is determined by the sign of D_0 and the transition between them marks a Hopf bifurcation.

VI. APOLAR AND CHIRAL FORCE DENSITY

We now discuss the effect of a chiral (but apolar) stress $\bar{z}_c[\nabla \times (\nabla\psi\nabla\psi)]^S$. In terms of the displacement field, the force density associated with this stress is $\zeta_c \nabla^2 \nabla \times \mathbf{u}_\perp$, with $\zeta_c = 3\bar{z}_c {\psi_1^0}^2 q_s^2$. Upon including the effect of this stress, the displacement dynamics in Fourier space becomes

$$\partial_t \tilde{\mathbf{u}}_\perp = \left[\frac{\eta}{\Delta q^2} \left(\frac{q_z^2}{q^2} \mathbf{e}_l \mathbf{e}_l + \mathbf{e}_t \mathbf{e}_t \right) - \frac{\nu_o q_z^2}{\Delta q^4} \boldsymbol{\epsilon} \right] \cdot \tilde{\boldsymbol{\mathcal{F}}}^e - \left[\frac{\eta q_z q_\perp}{\Delta q^4} \mathbf{e}_l + \frac{\nu_o q_z q_\perp}{\Delta q^4} \mathbf{e}_t \right] \big(\hat{\mathbf{z}} \cdot \tilde{\boldsymbol{\mathcal{F}}}^e \big). \tag{47}$$

where

$$\tilde{\boldsymbol{\mathcal{F}}}^e = - \Big[\big(\bar{\mu} q_\perp^2 + \zeta q^2 \big) \big(\mathbf{e}_l \mathbf{e}_l + \mathbf{e}_t \mathbf{e}_t \big) + (\bar{\mu} + \bar{\lambda}) q_\perp^2 \, \mathbf{e}_l \mathbf{e}_l + \big(\zeta_{pc} q_\perp^2 - i\zeta_c q^2 q_z \big) \boldsymbol{\epsilon} + i\zeta_c q^2 q_\perp \, \hat{\mathbf{z}} \mathbf{e}_t \Big] \cdot \tilde{\mathbf{u}}_\perp. \tag{48}$$

The dynamical matrix can still be written as in (41), but now with the following definitions:

$$D_0 = -\frac{\eta}{2\Delta q^4} \Big[\big(\bar{\mu} q_\perp^2 + \zeta q^2 \big) \big(2q_z^2 + q_\perp^2 \big) + (\bar{\mu} + \bar{\lambda}) q_z^2 q_\perp^2 \Big] - \frac{\nu_o [\zeta_{pc} q_z^2 q_\perp^2 - i\zeta_c q_z q^2 (q_z^2 + q^2)]}{\Delta q^4}, \tag{49}$$

$$D_{\rm r} = \frac{\eta[\zeta_{pc}(2q_z^2 + q_\perp^2) q_\perp^2 - 2i\zeta_c q^4 q_z]}{2\Delta q^4} - \frac{\nu_o q_z^2}{2\Delta q^4} \big[2\big(\bar{\mu} q_\perp^2 + \zeta q^2 \big) + (\bar{\mu} + \bar{\lambda}) q_\perp^2 \big], \tag{50}$$

$$D_+ = \frac{\eta q_\perp^2}{2\Delta q^4} \big[\bar{\mu} q_\perp^2 + \zeta q^2 - (\bar{\mu} + \bar{\lambda}) q_z^2 \big] - \frac{i\zeta_c \nu_o q_z q_\perp^2}{2\Delta q^2}, \tag{51}$$

$$D_\times = \frac{\eta \zeta_{pc} q_\perp^4}{2\Delta q^4} - \frac{\nu_o (\bar{\mu} + \bar{\lambda}) q_z^2 q_\perp^2}{2\Delta q^4}. \tag{52}$$

* sjk202@cam.ac.uk
† G.P.Alexander@warwick.ac.uk
‡ nyomaitra07@gmail.com
§ sriram@iisc.ac.in
[1] P. G. de Gennes and J. Prost, *The Physics of Liquid Crystals* (Clarendon, Oxford, 1993).
